# Association between Uric Acid and Bone Mineral Density in Postmenopausal Women with Type 2 Diabetes Mellitus in China: A Cross-Sectional Inpatient Study

**DOI:** 10.1155/2020/3982831

**Published:** 2020-06-13

**Authors:** Xin Zhao, Xiaofeng Yu, Xiaomei Zhang

**Affiliations:** Endocrinology Department, Peking University International Hospital, Beijing, China

## Abstract

**Objective:**

To analyze the association between uric acid levels and bone mineral density in postmenopausal women with type 2 diabetes mellitus.

**Methods:**

We retrospectively analyzed 262 postmenopausal women with type 2 diabetes mellitus, to assess uric acid levels and bone mineral density using the *T* score of dual-energy X-ray absorptiometry.

**Results:**

(1) Women in the osteoporosis group demonstrated higher uric acid levels and lower estimated glomerular filtration rate (*p* < 0.05, respectively). (2) Uric acid levels were positively correlated with the hip and lumbar spine bone mineral density and *T* score (*r* = 0.17, *p* < 0.05; *r* = 0.25, *p* < 0.05; *r* = 0.17, *p* < 0.05; and *r* = 0.28, *p* < 0.05, respectively). Meanwhile, there was a positive relation between estimated glomerular filtration rate and hip bone mineral density (*r* = 0.22, *p* < 0.05). (3) Logistic regression analysis showed that age, body mass index, and diabetic duration are independent risk factors for osteoporosis in postmenopausal women with type 2 diabetes mellitus. The level of estimated glomerular filtration rate and uric acid levels were not independent effect factors for osteoporosis in menopausal women.

**Conclusion:**

Uric acid levels are neither a protective factor nor a risk factor for osteoporosis in women with type 2 diabetes mellitus.

## 1. Introduction

With continuously changing modern lifestyle and increase in the number of aging individuals, comorbidities such as type 2 diabetes mellitus (T2DM) and osteoporosis (OP), which gradually increase in incidence with age, have become common health problems [1, 2]. Higher blood glucose levels in T2DM patients increase the risk of diabetic complications, such as increased risk of brittle fracture [3]. Studies have shown that the changes in bone infrastructure in T2DM patients are due to multifactorial causes and manifest as decreased, increased, or normal bone mass. The bone mineral density (BMD) of T2DM patients is higher than that of nondiabetic people; however, the risk of fracture in T2DM is also significantly higher in T2DM patients [4–6]. In postmenopausal women with T2DM, there are disorders of glucose, lipid, and uric acid (UA) metabolism and bone metabolism, and the risk of osteoporosis is significantly increased. As an end product of purine metabolism, UA is an important endogenous antioxidant. A large number of studies have shown that UA has certain protective effects on a variety of diseases caused by high oxidative stress, including osteoporosis [7], so it is generally considered that UA is a protective factor of osteoporosis. However, hyperuricemia is a risk factor for insulin resistance and diabetes. Hyperuricemia can aggravate the progress of diabetes; hyperglycemia can also lead to bone fragility [8], so hyperuricemia can indirectly accelerate bone loss in T2DM patients. Therefore, whether the increase of UA is still related to the BMD is worth exploring. The purpose of this study is to explore the correlation between UA and BMD and bone metabolism indices in postmenopausal women with type 2 diabetes in China, so as to provide theoretical basis for clinical prevention and treatment of osteoporosis.

## 2. Methodology

### 2.1. Participants

In this retrospective study, 262 postmenopausal women with T2DM who were hospitalized in Peking University International Hospital endocrinology department from January 2017 to December 2019 were analyzed. The average age of the participants was 63.65 ± 7.90 years (50-80 years), and the average duration of T2DM was 11.61 ± 6.94 years. All subjects met the T2DM diagnostic criteria of the World Health Organization (WHO) in 1999 [9]. The exclusion criteria included (1) other type of diabetes mellitus; (2) nonphysiological menopausal women; (3) long-term use of drugs that affect bone metabolism; (4) patients with a history of primary or secondary bone cancer; (5) patients who have used OP drugs (estrogen, bisphosphonate, active vitamin D, etc.); and (6) patients who have previously been diagnosed with hyperuricemia and have taken hypouricemia drug (allopurinol, benzbromarone, etc.).

### 2.2. Methods

#### 2.2.1. General Conditions

*(1) Basic Information Collected*. All participants' age, date of birth, diabetic duration, menopausal years, diabetes complications, including diabetic nephropathy, diabetic neuropathy, and diabetic retinopathy, and types of antidiabetic drugs were collected and recorded.

*(2) Height and Weight Measurement*. All participants were asked to take off their shoes and socks and wear light and thin clothes, following which height (cm) and weight (kg) were measured with measuring instrument, and body mass index (BMI) was obtained according to the formula weight/height^2^ (kg/m^2^). Blood pressure including diastolic blood pressure (DBP) and systolic blood pressure (SBP) was measured in all participants.

#### 2.2.2. Laboratory Measurement

All subjects were asked to fast for at least 8 hrs, and venous blood samples were collected in the morning. Chemiluminescence method was then used to test blood glucose and blood lipid profile. Other biochemical indices of the participants were then determined. High-pressure liquid chromatography (HPLC) was used to test glycosylated hemoglobin (HbAlc) level. The tests were carried out in the biochemical laboratory of Peking University International Hospital.

Laboratory measurements included fasting blood glucose (FBG), serum creatinine (sCr), glycosylated hemoglobin (HbA1c), calcium (Ca), uric acid (UA), low-density lipoprotein cholesterol (LDL-C), total cholesterol (TC), triglyceride (TG), high-density lipoprotein cholesterol (HDL-C), parathyroid hormone (PTH), osteocalcin (OC), beta C-terminal telopeptide (*β*-CTX), procollagen 1 intact N-terminal (P1NP), and 25-hydroxyvitamin D (25(OH)D). The glomerular filtration rates (eGFRs) were estimated according to the sCr level.

sCr < 0.7 mg/dl: eGFRCKD‐EPI‐ASIA = 141 × (sCr/0.7)^−0.329^ × 0.993^age^ × 1.049.

SCr > 0.7 mg/dl: eGFRCKD‐EPI‐ASIA = 141 × (sCr/0.7)^−1.209^ × 0.993^age^ × 1.049.

#### 2.2.3. BMD Measurement

Dual-energy X-ray absorptiometry (DXA) was used to detect the bone mineral density of the hip and lumbar spine of the participants. The *T* score was automatically generated by the computing system according to the BMD of each part by software (Hologic, USA) used in the laboratory of Peking University International Hospital. Participants were divided into 3 groups according to *T* score: the normal group (*T* score > 1.0): 41 women, the osteopenia group (–1.0 ≥ *T* score ≥ –2.5): 122 women, and the osteoporosis group (*T* score < −2.5): 99 women.

#### 2.2.4. FRAX Score

According to the consensus of Chinese experts on fracture risk management of diabetic patients, FRAX score was determined for patients with T2DM, which was accessed at https://www.sheffield.ac.uk/frax/index. Under FRAX fracture risk assessment system, “Asia China mainland” mode was selected. The “calculate” button then provided the main OP fracture probability (PMOF) and hip fracture probability (PHF) within ten years.

### 2.3. Statistical Methods

All data were processed by SPSS 25.0. Normal distribution data were shown as mean standard deviation (*x* ± *s*), and nonnormal distribution data were shown as mean median and quartile spacing. When data was normally distributed and variance was homogeneous, variance analysis was used for comparison among groups. When data was not normally distributed, variance analysis such as Kruskal Wallis test was used for comparison among multiple groups; Pearson correlation analysis and Spearman correlation analysis were used for correlation analysis; logistic regression method was used for analysis of the main influencing factors of OP in postmenopausal women with T2DM, and *p* < 0.05 was used for statistical significance.

## 3. Results

### 3.1. General Characteristics, Biochemical Indices, BMD, and Bone Metabolism Markers among the 3 Groups

Compared with the other two groups, the patients in the OP group were older, had lower BMI, and had been diabetic and menopausal for longer duration (*p* < 0.05). Compared with the other two groups, PMOF and PHF in the OP group were significantly different (*p* < 0.05). eGFR in the OP group was lower than that in the other two groups whereas the level of UA in the OP group was higher than that in the other two groups (*p* < 0.05, respectively). There was no significant difference in blood pressure and blood lipid levels among the normal group, the osteopenia group, and the osteoporosis group (*p* > 0.05, respectively). There was no significant difference among the three groups on incidence rate of diabetic nephropathy, diabetic neuropathy, and diabetic retinopathy (*p* > 0.05, respectively). There was no significant difference on types of antidiabetic drugs among the three groups (*p* > 0.05, respectively). Upon comparison of bone metabolism markers among the three groups, the OC level was the highest and 25(OH)D level was the lowest in the normal group, while the OC level was the lowest and 25(OH)D level was the highest in the osteoporosis group, and the difference was statistically significant (*p* < 0.05, respectively) (shown in Table 1).

### 3.2. Correlation Analysis between BMD and Age, BMI, Diabetes Duration, Menopausal Year, HbA1c, Glucose and Blood Lipid Profile, UA, eGFR, and Other Biochemical Indices in Postmenopausal Women with T2DM

**Figure 1:**
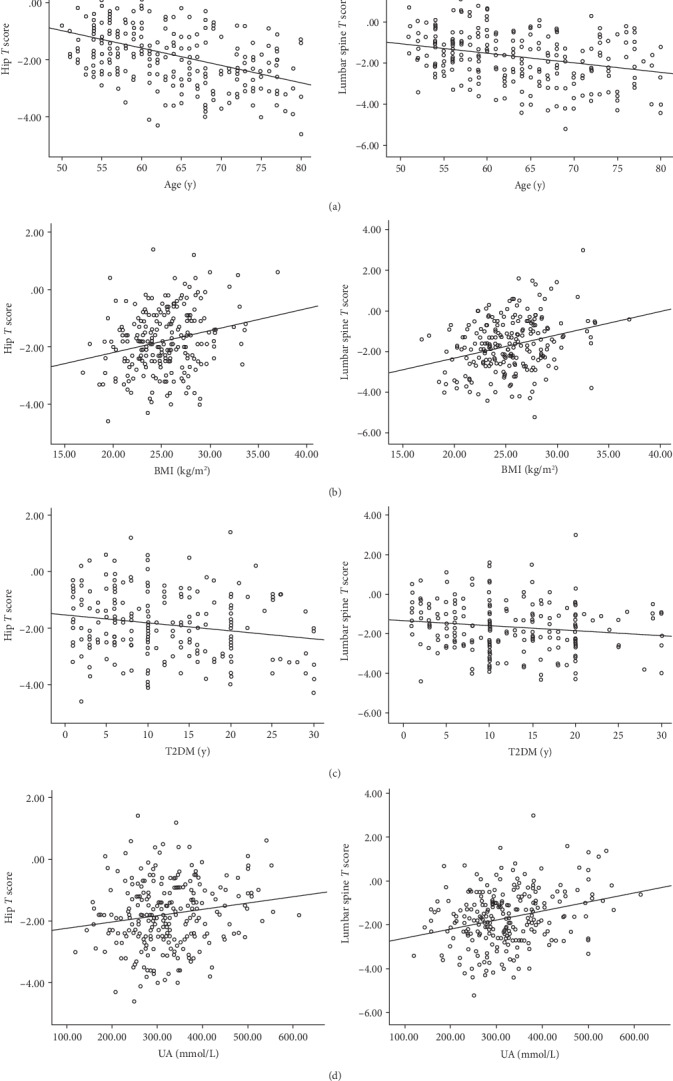
Comparison of the relation between age, BMI, and T2DM duration and hip and lumbar spine *T* score, respectively. (a) Correlation analysis revealed that age was negatively correlated with hip and lumbar spine *T* score (*r* = −0.44, *p* < 0.05; *r* = −0.28, *p* < 0.05, respectively). (b) Correlation analysis revealed that BMI was positively correlated with hip *T* score and lumbar spine *T* score (*r* = 0.17, *p* < 0.05; *r* = 0.24, *p* < 0.05, respectively). (c) Correlation analysis revealed that BMI was negatively correlated with hip *T* score and lumbar spine *T* score (*r* = −0.21, *p* < 0.05; *r* = −0.19, *p* < 0.05, respectively). (d) Correlation analysis revealed that UA was positively correlated with hip *T* score and lumbar spine *T* score (*r* = 0.17, *p* < 0.05; *r* = 0.28, *p* < 0.05, respectively).

Among the three groups, age, diabetes duration, and menopausal year were negatively correlated with BMD (hip and lumbar spine) as well as the *T* score (hip and lumbar spine) (*p* < 0.05, respectively). On the other hand, BMI was positively correlated with BMD (hip and lumbar spine) as well as the *T* score (hip and lumbar spine) (*p* < 0.05, respectively). There was a positive correlation between the level of UA and BMD and *T* score (*p* < 0.05, respectively). Meanwhile, eGFR level was positively correlated with hip BMD (*r* = 0.22, *p* < 0.05) (shown in Table 2 and Figure 1).

### 3.3. Binary Logistic Regression Analysis of the Relationship between UA and Osteoporosis in Postmenopausal Women with T2DM

After adjusting the blood pressure, blood lipid profile, blood glucose, calcium, and PTH indices, eGFR and UA were not the independent factors for OP in postmenopausal women with T2DM; however, the age, lower BMI, and T2DM duration were independent risk factors (shown in Table 3).

## 4. Discussion

The relationship between T2DM and OP has been widely studied; however, the results are still controversial. Although T2DM patients have normal or even increased BMD, the risk of fracture is higher in T2DM patients than in nondiabetics. Because of this contradiction [10–12], screening for risk factors of OP in T2DM patients as early as possible is the key mechanism of OP prevention and treatment.

Currently, BMD is the gold standard used to evaluate bone mass and diagnose OP in the clinic. Although BMD is the most important factor to predict fracture risk, many brittle fractures in T2DM patients occur in individuals with *T* score higher than -2.5 as seen in clinical practice. Some studies [2] have proposed that increased fracture risk in T2DM patients results from various causes, including increased disease duration, poor blood glucose control, falls caused by hypoglycemia, decreased bone mass, impaired bone mass, and adverse drug reactions. In this study, T2DM duration is an independent risk factor for OP, suggesting that the incidence of OP in T2DM is complex and that the causes are multifactorial.

T2DM may affect bone health through a variety of complex ways. (1) Insulin resistance [13, 14]: insulin resistance is an important factor causing dysfunction of osteoblasts and osteoclasts activity. In addition, high blood glucose level can induce cell glycotoxicity, leading to osteoblast apoptosis. (2) Advanced glycation end products (AGEs): one of the inducers of brittle fracture in T2DM patients is age, with older age increasing the risk of brittle fracture in T2DM patients by inducing abnormal collagen arrangement [15, 16]. (3) Calcium loss in urine and vitamin D deficiency: diabetes caused by hyperglycemia results in an increase in calcium levels in the urine and the decrease in calcium level *in vivo*, leading to apoptosis of osteoblasts and the accelerated bone loss. (4) Diabetic complications: diabetic microvascular complications reduce blood supply to bone tissue, leading to bone loss [17]. (5) Use of some hypoglycemic drugs, such as insulin, thiazolidinediones, and sodium-glucose cotransporter 2 (SGLT-2), is related to bone loss and increased risk of fracture, especially in women [18].

This study screened the risk factors of OP in postmenopausal women with T2DM. Research shows that in the normal population, aging, menopause, and lower BMI are the independent risk factors of OP, which has been widely recognized [19]. This result was further confirmed in the postmenopausal women with T2DM in this study. As previously noted [20], this study also found that the increased T2DM duration is an independent factor for postmenopausal women. All of these findings indicate that T2DM patients who are older, with lower BMI, and with longer T2DM duration and menopausal year may have lower BMD and, therefore, higher incidence of OP and greater risk of fracture.

In recent years, studies [21] have shown that UA can promote the proliferation and osteogenic differentiation of human mesenchymal stem cells. UA is closely related to oxidative stress in the human body, and the increase of oxidative stress or the decrease of antioxidants will reduce the level of BMD. Whether UA is a protective factor or a risk factor of OP is controversial. At present, it is believed that UA has double effects on the body. The physiological concentration of UA has a protective effect on the stability of bone mass, while the excessive UA has the opposite effect. The mechanism of the increase of BMD induced by UA may be as follows: oxidative stress can inhibit the differentiation of osteoblasts and induce the death of osteoblasts. As a reducing substance, UA can prevent the production of reactive oxygen species in osteoblasts and stimulate the differentiation of osteoblasts, thus increasing bone formation [22]; UA can also inhibit the generation of osteoclasts, reduce the production of oxygen free radicals by osteoclast precursors, and reduce bone absorption. Foreign scholars [23] believe that there is a positive correlation between BMD and UA, and when UA is between 4 and 4.99 mg/dl, it reduces the risk of osteoporosis. Similarly, Ishii et al. [24] found that the level of hyperuricemia in the physiological range was linearly related to the increase of lumbar BMD in Japanese postmenopausal women, but whether there was still a positive correlation between the two indices in the hyperuricemia range is questioned. A larger population study believes that the increase of uric acid level is protective for bone density and bone strength [24, 25]. However, some studies have suggested that hyperuricemia is a risk factor for OP due to the role of inflammatory factors and the involvement of oxidative stress response [26, 27]. In this study, UA and eGFR were found to have positive correlation with bone mineral density and *T* score, which has previously been reported in patients without T2DM [28]. Due to the influence of metabolic indicators such as blood glucose, blood lipid, and blood pressure, the direct effect of UA on OP may not be found. In this study, after adjusting for BMI, age, blood pressure, blood glucose, blood lipid profile, and other factors, UA and eGFR were not found to be independent risk or protective factors of OP in postmenopausal women with T2DM. This finding suggests that the correlation between UA and eGFR and BMD might be due to the influence of BMI and metabolism index. After excluding the confounding factors, UA and eGFR were not found to be independent factors of OP in women with T2DM. In addition, in this study, the subjects were all inpatients. The level of blood glucose was higher than that of outpatients (the mean HbA1c level was 8.3-8.8%). Therefore, the risk of hypoglycemia was little, so the subjects are not fragile patients.

In this study, there was no significant difference among the three groups in the occurrence of complications and the application of hypoglycemic drugs, so the complications of diabetes and the interference of hypoglycemic drugs on the results were excluded as much as possible.

However, there are a few limitations in this study. The sample size needs to be larger to better assess the risk factors of OP in T2DM. Also, whether UA is a protective factor or a risk factor of OP in patients with T2DM could not be clarified due to the complexity of pathophysiological mechanism and the interference of multiple metabolic indicators. Therefore, further longitudinal research and large-sample epidemiological data is needed to confirm any finding.

More and more studies have shown that T2DM is a clinical risk factor that leads to increase in fracture. Many commonly used clinical indices such as the effect of UA level on osteoporosis have not been confirmed. In postmenopausal patients with type 2 diabetes in our study, uric acid levels do not influence either positively or negatively bone mineral density. Therefore, it is of great clinical significance to find more risk or protective factors of OP for preventing the occurrence of fracture in such patients.

## Figures and Tables

**Table 1:** Comparison of general characteristics, diabetic complication, biochemical indices, BMD, and bone metabolism markers among the three groups.

Index	Normal group (*n* = 41)	Osteopenia group (*n* = 122)	Osteoporosis group (*n* = 99)	*F* (*X*^2^)	*p*
Age (year)	59.71 ± 6.06	62.10 ± 7.85^a^	67.20 ± 7.27^a,b^	9.93	<0.05
BMI (kg/m^2^)	26.23 ± 3.50	25.76 ± 3.63^a^	24.96 ± 3.77^a,b^	3.26	<0.05
Diabetes duration (year)	10.53 ± 6.83	12.03 ± 6.98	13.82 ± 6.96^a,b^	3.18	<0.05
Menopausal year (year)	11.15 ± 4.12	15.67 ± 5.28^a^	19.17 ± 6.93^a,b^	2.87	<0.05
SBP (mmHg)	139.34 ± 17.01	133.82 ± 16.82	137.12 ± 18.85	1.86	0.16
DBP (mmHg)	79.32 ± 7.49	77.09 ± 10.36	77.14 ± 11.91	0.75	0.47
PMOF (%)	2.5 (2.0-2.7)	3.6 (3.2-4.3)^a^	7.2 (5.8-9.7)^a,b^	2.96	<0.05
PHF (%)	0.3 (0.2-0.5)	1.0 (0.6-1.4)^a^	3.8 (1.8-5.3)^a,b^	2.45	<0.05
HbA1c (%)	8.45 ± 2.09	8.88 ± 1.91	8.29 ± 1.89	2.38	0.10
FBG (mmol/l)	8.53 ± 3.21	9.07 ± 3.83	8.07 ± 2.79	0.20	0.82
TC (mmol/l)	4.37 ± 1.14	4.44 ± 1.09	4.56 ± 1.36	0.42	0.66
TG (mmol/l)	1.97 ± 1.33	2.13 ± 1.62	1.83 ± 1.44	0.98	0.38
LDL-C (mmol/l)	2.51 ± 0.80	2.61 ± 1.06	2.78 ± 1.03	1.21	0.30
HDL-C (mmol/l)	1.04 ± 0.25	1.06 ± 0.27	1.10 ± 0.27	1.13	0.32
UA (*μ*mol/l)	336.20 ± 92.41	324.84 ± 93.15^a^	312.67 ± 74.01^a,b^	3.26	<0.05
eGFR (ml/min/1.73^2^)	97.25 ± 13.29	89.36 ± 19.85^a^	86.78 ± 20.25^a,b^	4.23	<0.05
Ca (mmol/l)	2.33 ± 0.09	2.32 ± 0.12	2.30 ± 0.08	1.34	0.27
PTH (pmol/l)	36.63 ± 13.60	38.63 ± 14.81	40.74 ± 15.78	1.16	0.32
Lumbar BMD (g/cm^2^)	1.05 ± 0.17	0.89 ± 0.11^a^	0.72 ± 0.14^a,b^	100.71	<0.05
Hip BMD (g/cm^2^)	0.82 ± 0.06	0.67 ± 0.07^a^	0.55 ± 0.09^a,b^	194.02	<0.05
OC (ng/ml)	17.14 ± 9.75	14.51 ± 6.89^a^	12.80 ± 5.05^a,b^	2.79	<0.05
*β*-CTX (ng/ml)	0.35 ± 0.23	0.43 ± 0.24	0.45 ± 0.27	2.24	0.11
P1NP (ng/ml)	42.56 ± 26.90	44.76 ± 24.10	50.62 ± 25.53	2.07	0.13
25(OH)D (ng/ml)	18.29 ± 4.99	16.10 ± 5.47^a^	13.89 ± 5.85^a,b^	2.99	<0.05
Complications					
Nephropathy (%)	7 (17.07%)	25 (20.49%)	17 (17.17%)	0.48	0.79
Neuropathy (%)	5 (12.20%)	13 (10.66%)	13 (13.13%)	0.33	0.85
Retinopathy (%)	6 (14.63%)	15 (12.30%)	10 (10.10%)	0.62	0.73
Types of antidiabetic drugs					
Metformin (%)	33 (80.49%)	89 (72.95%)	66 (66.67%)	2.89	0.24
SU (%)	10 (24.39%)	24 (19.67%)	22 (22.22%)	0.47	0.79
*α*-Glycosidase inhibitors (%)	10 (24.39%)	44 (36.07%)	37 (37.37%)	2.33	0.31
SGLT-2 inhibitor (%)	1 (2.44%)	2 (1.64%)	1 (1.01%)	0.41	0.81
TZD (%)	0	0	0	—	—
GLP-1 receptor agonist (%)	2 (4.88%)	4 (3.28%)	3 (3.03%)	0.32	0.85
DDP-4 inhibitor (%)	31 (75.61%)	78 (63.93%)	65 (65.66%)	1.92	0.38
Insulin (%)	12 (29.27%)	33 (27.05%)	19 (19.19%)	2.44	0.29

Note: ^a^*p* < 0.05 compared with the normal group; ^b^*p* < 0.05 compared with the osteopenia group. SBP: systolic blood pressure; DBP: diastolic blood pressure; BMI: body mass index; FBG: fasting blood glucose; HbA1c: glycosylated hemoglobin; UA: uric acid; Ca: calcium; TC: total cholesterol; TG: triglyceride; LDL-C: low-density lipoprotein cholesterol; HDL-C: high-density lipoprotein cholesterol; PTH: parathyroid hormone; eGFR: glomerular filtration rate; BMD: bone mineral density; PMOF: probability of a major osteoporotic fracture; PHF: probability of hip fracture; SU: sulfonylurea; SGLT-2: sodium-glucose cotransporter 2; TZD: thiazolidinedione; GLP-1: glucagon-like peptide 1; DDP-4; dipeptidyl peptidase 4.

**Table 2:** Correlation analysis between BMD and general conditions and biochemical indices in postmenopausal women with T2DM.

Index	Hip BMD		Lumbar spine BMD	
	*r*	*p*	*r*	*p*
Age (year)	-0.44	<0.05	-0.28	<0.05
BMI (kg/m^2^)	0.16	<0.05	0.20	<0.05
Diabetes duration (year)	-0.18	<0.05	-0.25	<0.05
Menopausal year (year)	-0.28	<0.05	-0.33	<0.05
HbA1c (%)	-0.01	0.94	0.01	0.87
FBG (mmol/l)	-0.06	0.38	0.07	0.40
TC (mmol/l)	0.02	0.76	-0.01	0.95
TG (mmol/l)	0.06	0.41	0.01	0.89
LDL-C (mmol/l)	0.05	0.48	0.11	0.18
HDL-C (mmol/l)	0.02	0.74	-0.02	0.69
UA (*μ*mol/l)	0.17	<0.05	0.25	<0.05
eGFR (ml/min/1.73^2^)	0.22	<0.05	0.07	0.30
Ca (mmol/l)	0.14	0.06	0.08	0.23
PTH	0.12	0.08	0.18	0.24

Note: SBP: systolic blood pressure; DBP: diastolic blood pressure; BMI: body mass index; FBG: fasting blood glucose; HbA1c: glycosylated hemoglobin; UA: uric acid; Ca: calcium; TC: total cholesterol; TG: triglyceride; LDL-C: low-density lipoprotein cholesterol; HDL-C: high-density lipoprotein cholesterol; PTH: parathyroid hormone; eGFR: glomerular filtration rate; BMD: bone mineral density.

**Table 3:** Binary logistic regression analysis of the relationship between UA and osteoporosis in postmenopausal women with T2DM.

Index	OP		
	*β*st	OR (95% CI)	*p*
Age (year)	0.14	1.15 (1.08, 1.22)	<0.05
BMI (kg/m^2^)	-0.16	0.86 (0.77, 0.96)	<0.05
Diabetes duration (year)	0.05	1.05 (1.01, 1.11)	<0.05
eGFR (ml/min/1.73^2^)	0.00	1.00 (0.98, 1.03)	0.78
UA (*μ*mol/l)	-0.01	1.00 (0.99, 1.01)	0.81

Note: BMI: body mass index; UA: uric acid; eGFR: glomerular filtration rate.

## Data Availability

The data used to support the findings of this study are available from the corresponding author upon request.
